# Differential CARM1 expression in prostate and colorectal cancers

**DOI:** 10.1186/1471-2407-10-197

**Published:** 2010-05-13

**Authors:** Young-Rang Kim, Byung Kook Lee, Ra-Young Park, Nguyen Thi Xuan Nguyen, Jeong A Bae, Dong Deuk Kwon, Chaeyong Jung

**Affiliations:** 1 Department of Anatomy, Chonnam National University Medical School, Gwangju, Korea; 2 Department of Emergency Medicine, Chonnam National University Medical School, Gwangju, Korea; 3 Medical Research Center for Gene Regulation, Chonnam National University, Gwangju, Korea; 4 Department of Urology, Chonnam National University Medical School, Gwangju, Korea; 5 Research Institute of Medical Sciences, Chonnam National University, Gwangju, Korea

## Abstract

**Background:**

Coactivator-associated arginine methyltransferase 1 (CARM1) functions as a transcriptional coactivator of androgen receptor (AR)-mediated signaling. Correspondingly, overexpression of CARM1 has been associated with the development of prostate cancer (PCa) and its progression to androgen-independent PCa. In our preliminary study, however, the promoting effects of CARM1, with regard to androgen-stimulated AR target gene expression were minimal. These results suggested that the AR target gene expression associated with CARM1 may result primarily from non-hormone dependent activity. The goal of this study was to confirm the pattern of expression of CARM1 in human tumors and determine the mechanism of action in CARM1 overexpressed tumors.

**Methods:**

Tissue microarray was used to determine the pattern of expression of CARM1 in human cancers by immunohistochemistry. CARM1 expression was also evaluated in prostate and colorectal surgical specimens and the clinical records of all cases were reviewed. In addition, a reporter transcription assay using the prostate-specific antigen (PSA) promoter was used to identify the signaling pathways involved in non-hormone-mediated signal activation associated with CARM1.

**Results:**

The tissue microarray showed that CARM1 was particularly overexpressed in the colorectal cancers while CARM1 expression was not prevalent in the prostate and breast cancers. Further studies using surgical specimens demonstrated that CARM1 was highly overexpressed in 75% of colorectal cancers (49 out of 65) but not in the androgen-independent PCa. In addition, CARM1's coactivating effect on the entire PSA promoter was very limited in both androgen-dependent and androgen-independent PCa cells. These results suggest that there are other factors associated with CARM1 expression in PSA regulation. Indeed, CARM1 significantly regulated both p53 and NF-κB target gene transcription.

**Conclusions:**

The results of this study suggest that, in addition to its role in activation of steroid receptors, CARM1 functions as a transcriptional modulator by altering the activity of many transcriptional factors, especially with regard to androgen independent PCa and colorectal cancers.

## Background

CARM1 is a protein with arginine-specific histone methyltransferase activity [1]; it initially was described as a transcriptional activator of the p160 family of nuclear receptor-associated proteins (Src-1, GRIP1/TIF2/Src-2, ACTR/AIB1/SRC-3)[2-5]. The p160 coactivators act as primary coactivators through direct binding to the C-terminal region of nuclear receptors in a ligand-dependent manner. The p160 coactivators are involved in transcriptional activation by bringing secondary activators with them to the promoter. While p300 and CBP bind to the activation domain (AD) 1 of p160 [6], CARM1 binds to AD2 [1]. Ultimately, CARM1 promotes nuclear receptor activity through the formation of a tertiary complex of CARM1, p300/CBP, and p160 [3,5]. CARM1 has been shown to be a molecular switch that controls multiple classes of gene-specific transcription factors, including p53, NF-κB, LEF1/TCF4, and E2Fs [7-10]. Therefore, CARM1 appears to play a pleiotropic role in cell proliferation and survival.

CARM1 has primarily been studied in association with nuclear receptors, including the estrogen receptors (ER) and androgen receptors (AR). Cancers of the breast and prostate have been shown to overexpress CARM1 [7,11-13]. Overexpression of CARM1 has also been suggested in androgen-independent prostate cancers (PCa) [12,13]. The altered expression of CARM1 could provide a growth advantage for the PCa cells by enhancing the AR transactivation function and target gene activation. Here, we show that CARM1 overexpression was surprisingly prevalent in colorectal cancers while only a fraction of tumors from the breast and prostate overexpressed CARM1. Accordingly, we demonstrate that CARM1-mediated AR target gene expression, namely prostate-specific antigen, was minimal. These findings suggest that CARM1's role is mainly linked to the p53 and NF-κB response in non-nuclear receptor mediated cancer development similar to colorectal cancers and androgen-independent progression of PCa.

## Methods

### Plasmids and Reagents

pSG5-CARM1 was generously provided by Dr. Michael Stallcup; p61-luc contains the entire PSA promoter as previously described [14]. The pGL-ARE4-Luc contains a synthetic TATA and four tandem copies of androgen response elements (AREs), from the murine mammary tumor virus promoter, in the context of the pGL3 backbone (Promega). A human AR expression vector, pARO, was a gift from Dr. Jan Trapman. The p53-luc containing two copies of p53 response elements was purchased from BD biosciences. The p21-luc was provided by Dr. Bert Vogelstein. The pE2F1-luc was received from Dr. Chihuei Wang (Kaohsiung Medical University, Taiwan). The pTCF4RE-luc contains four copies of the TCF-4 response elements derived from the cyclin D1 promoter. pBV-cmyc-luc was generously provided by Dr. Bert Vogelstein. pRC/RSV-p300 was received from Dr. Richard Goodman (The Cleveland Clinic). CARM1 S229E mutants were made on pSG5-CARM1 backbone using QuickChange Lightning Site-Directed Mutagenesis kit (Stratagene, La Jolla, CA, USA)[15]. Polyclonal antibodies to CARM1 were received from Dr. Meei-Huey Jeng (Indiana University) and monoclonal antibodies to β-actin were purchased from Santa Cruz Biotechnology. Synthetic testosterone, R1881, was purchased from NEN Life Science (Boston, MA, USA) and used at a final concentration of 10 nM.

### Cell Culture

Human prostate cell lines, including P69, LNCaP, C4-2, CWR22RV, PC3 and DU145 were routinely cultured in RPMI media (Invitrogen) supplemented with 5% FBS at 37°C in an atmosphere containing 5% CO_2 _as described previously [16]. MDA PCa 2b prostate cancer cells were grown in BRFF-HPC1 medium (Athena Environmental Sciences, Inc., Baltimore, MD, USA) with 20% FBS. CV-1 monkey kidney cells, MDA-MB-231 human breast cancer cells, and caco-2 colon cancer cells were maintained in DMEM containing 10% FBS. All cultures were fed with fresh medium every 3-4 days.

### Real-Time RT-PCR

Total RNA extraction from selected cultured cells was performed as previously described [17]. RNA from MDA231 cells was used for standardizing the expression levels of CARM1 and GAPDH. Fifty nanograms (ng) of total RNA from each sample were used to detect real-time RT-PCR (QRT-PCR) products with Taqman probes and an ABI PRISM 7700 sequence detection system (PE Applied Biosystems, Foster City, CA, USA). PCR cycling conditions for all of the samples were as follows: 30 min at 48°C for reverse transcription; 10 min at 95°C for AmpliTaq Gold activation; and 40 cycles for the melting (95°C, 15 s) and annealing/extension (60°C, 1 min) steps. CARM1 and GAPDH primers and probes for QRT-PCR were designed using the PRIMER Express program (PE Applied Biosystems). The sequences of the CARM1 primers and probe were as follows: forward, 5'-ttgatgttggctgtggctctgg-3'; reverse, 5'-atgggctccgagatgatgatgtcc-3'; probe, 5'-FAM-caacctgacggaccgcatcgtg-TAMRA-3'. The sequences of the GAPDH primers and probe were as follows: forward, 5'-gaaggtgaaggtcggagtc-3'; reverse, 5'-gaagatggtgatgggatttc-3'; probe, 5'-VIC-caagcttcccgttctcagcc-TAMRA-3'. All QRT-PCR experiments were performed twice in duplicate in one 96-well plate. Using the comparative C_T _method (PE Applied Biosystems), the resulting Ct values were converted to picogram(pg) quantities according to each standard curve. Then, the quantity of CARM1 was normalized to GAPDH and subtracted from no reverse transcriptase controls. This value was then averaged for each duplicate.

### Transient Transfections

Approximately 1 × 10^5 ^cells were plated in a 24-well plate 16 hours before the transfection. To determine the hormone effect, the cells were grown under 5% charcoal dextran-treated (CDT) FBS for three days before the transfection. The transfections were carried out using the Lipofectamine 2000 (Invitrogen) with 0.1 μg of reporter, 0.1 μg of test plasmid, and 2 ng renilla as described by the manufacturer's protocol. Six hours after transfection, the cells were washed and fed with medium containing 5% CDT-FBS. If needed, the cells were treated with either R1881 synthetic androgen or ethanol. After 36 hours, the cells were washed with PBS, lysed with 100 μl of passive lysis buffer, and assayed for luciferase activity as relative light units using the Dual Luciferase assay system (Promega). The transfection experiments were performed in triplicate and the results are reported as the mean ± S.D. The relative luciferase activity (RLU) was measured. When necessary, the RLU from the hormone-treated group was normalized by the non-hormone-treated group and the values represented as fold change.

### Immunohistochemistry

Multi-tumor tissue microarray slides were obtained from the Cooperative Human Tissue Network under the Tissue Array Research Program (TARP) of the National Cancer Institute, The National Institutes of Health, Bethesda, MD, USA. Human colorectal tumors were obtained from the Indiana University Tissue Bank. There were 65 tumors with matching normal tissues adjacent to the tumors. All tumors were formalin-fixed and paraffin-embedded. After deparaffinizing the tissues, slides were microwaved for 10 min, three times in 10 mM citrate buffer (pH 6.0) to retrieve the antigens. Then, endogenous peroxidase was removed by the treatment of the tissues with 0.3% H_2_O_2 _followed by avidine-biotin blocking. To inhibit nonspecific binding of antibodies, the slides were treated with 3% normal goat serum before the overnight incubation of the slides with anti-CARM1 antibodies. Then, the signals were amplified by the horseradish peroxidase-DAB detection method.

### Western Blot Assay

The cells were grown to 80% confluence in P60 culture dishes containing 5% FBS-T media. The cells were then lysed in protein extraction buffer (1× TBS, 1% NP-40, 0.5% sodium deoxycholate), 0.1% SDS and protease inhibitors. Twenty μg of total cell lysates were loaded onto a 10% Bis-Tris gel and separated using the electroporation system (Biorad). After the proteins were transferred to a PVDF membrane, primary antibodies were applied, followed by incubation with horse peroxidase-conjugated secondary antibodies. The blots were developed by the ECL detection system (Pierce).

### Chromatin Immunoprecipitation

Chromatin immunoprecipitation was performed as described [18] using antibodies against p53, NF-κB p65, or CARM1. The immunoprecipitated DNA was amplified by using specific primers as follows: p21 (ctcacatcctccttcttcag, cacacacagaatctgactccc), CCND1 (tcagggatggcttttggg, caacttcaacaaaactcccc).

## Results

### CARM1 was highly expressed in colorectal cancers, but not in breast and prostate cancers

**Figure 1:**
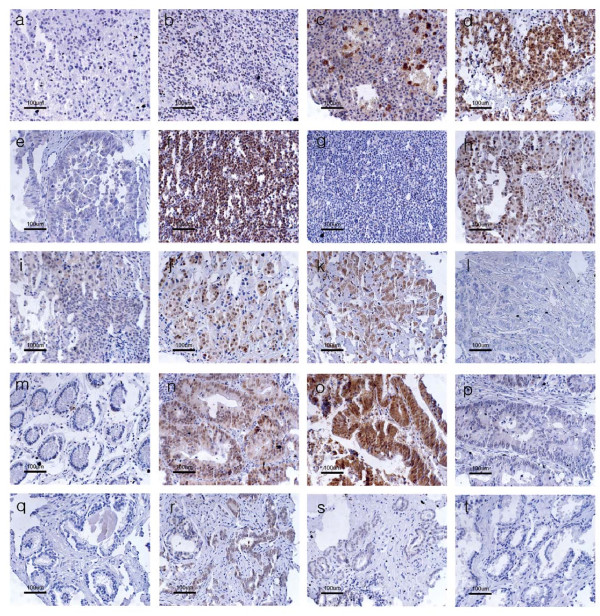
**Expression pattern of CARM1 in human tumor tissue array**. Immunohistochemical staining was performed on the tumor tissue arrays. a-b, brain tumor; c, melanoma; d-e, ovarian cancer; f-g, lymphoma; h-i, lung cancer; j-l, breast cancer; m, normal colon; n-p, colorectal cancer; q, normal prostate; r-t, prostate cancer. Scale bar represents 100 μm.

To determine the pattern of expression of CARM1 during tumorigenesis, tissue microarrays containing various tumors with several normal tissues were used to immunolocalize CARM1. Routine hematoxyline and eosine staining from two different types of tissue microarray slides identified a valid number of tumor tissues, including 35 melanomas, 76 lymphomas, 73 breast tumors, 109 prostate tumors, 103 colon tumors, 96 lung tumors, 35 brain tumors, and 35 ovarian tumors. The immunolocalized CARM1 was graded as 0-3 (0 as no expression; 1 as weak and focal expression; 2 as moderate and diffuse expression; 3 as strong and widespread expression). The immunostained slides were evaluated by two different investigators, including a pathologist, blinded to patient's clinical findings. Tumors with less than 5% CARM1 expression were considered negative regardless of the intensity described [19]. This approach has been previously reported to be reliable and reproducible by several groups [20-22]. The normal colon (Fig. 1m) and prostate (q) tissues generally showed no to very low, expression of CARM1. In addition, there was no to low expression of CARM1 in brain tumors (a-b) and melanomas (c). The melanomas showed only melanin granules. High or low expression of CARM1 was observed in ovarian cancers (d-e), lymphomas (f-g), lung cancers (h-i), breast cancers (j-l), colorectal cancers (n-p), and prostate cancers (r-t). Table 1 summarizes the CARM1-stained tumor scores. We arbitrarily, set a score of 2-3 as the overexpressed group compared to a score 0-1 as the standard. In contrast to our prediction that CARM1 would be highly expressed in steroid receptor-rich tumors, CARM1 was only overexpressed in a small fraction of these tumors (27% of breast tumors, 6% of prostate cancers, and 17% of ovarian cancers). In contrast to a previous report [12], overexpression of CARM1 in prostate cancers was not observed. Surprisingly, 68% of colorectal tumors overexpressed CARM1; however, overexpression was very limited in all other tumors.

**Table 1:** Score of CARM1 expression in human tumors

Tumor types	Score of CARM1 expression*					
	
	0	1	2	3	0-1**	2-3**
Brain tumor	22	10	3	0	32	3 (8%)
Melanoma	25	5	2	3	30	5 (14%)
Ovarian cancer	22	7	5	1	29	6 (17%)
Lymphoma	58	9	8	1	67	9 (12%)
Lung cancer	61	25	8	2	86	10 (10%)
Breast cancer	31	22	15	5	53	20 (27%)
Colorectal cancer	16	17	39	31	33	70 (68%)
Prostate cancer	85	17	5	2	102	7 (6%)

*, CARM1 expression was scored as followed: 0, no; 1, low; 2, moderate; 3, high

**, Scores were categorized by 0-1 as normal expression and 2-3 as overexpression

### CARM1 was not overexpressed in androgen-independent prostate cancer

**Figure 2:**
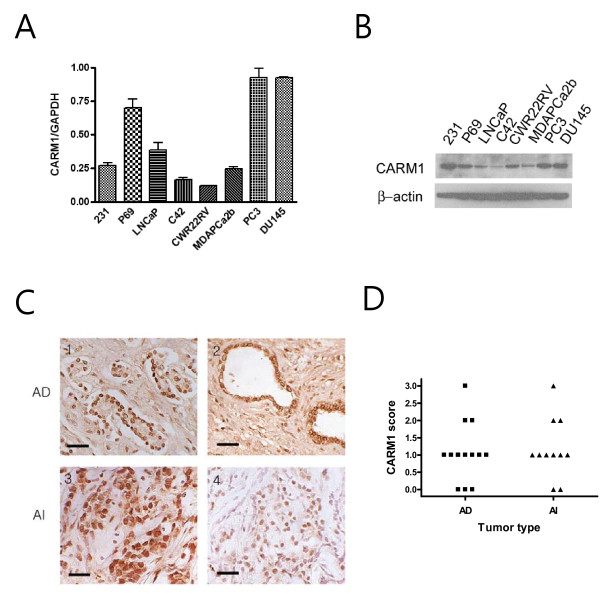
**Expression of CARM1 in prostate cancers**. *A*, Quantitative RT-PCR analysis demonstrated expression of CARM1 from the total RNA of various cultured cells; *B*, Western blot analysis of CARM1 expression in the indicated cells; *C*, Expression of CARM1 in androgen dependent (AD) tumors (1-2) and androgen-independent (AI) tumors (3-4). Scale bar represent 100 μm; *D*, Dot plot demonstration of CARM1 expression score in prostate tumors.

CARM1's overexpression during the development of prostate cancer continues to be debated. CARM1 has been reported to be overexpressed in androgen-resistant prostate cancers when compared to androgen-dependent cancers [12,13]. The results of this study showed that CARM1 overexpression was not generally observed using a prostate cancer tissue microarray. Next, we studied whether overexpression of CARM1 was present in androgen-resistant tumors. First, quantitative RT-PCR and Western blot analyses were performed to confirm the CARM1 expression level in various prostate cancers (Fig. 2A-B). P69 normal prostate cells and the human breast cancer cell line MDA-MB-231 were used to demonstrate the baseline expression of CARM1. Both RNA (A) and protein (B) levels of CARM1 were consistent on both analyses. The findings showed that CARM1 was generally underexpressed in prostate cancer cells excluding PC3 and DU145, AR-deficient cells. The CARM1 expression pattern was re-accessed with surgical or biopsied tumor specimens of patients with failed androgen ablation therapy and compared to tumors without PSA recurrence. Twenty-four clinical samples of hormone-dependent (n = 13) and hormone-refractory (n = 11) prostate carcinomas were successfully stained. As shown in Figure 2C, a variable amount of CARM1 was expressed in both androgen-dependent (AD) and androgen-independent (AI) tumors. In contrast to previous reports [12,13], however, CARM1 expression was not altered during androgen-independent progression of prostate cancer as shown by the dot-plot analysis (Fig. 2D).

### CARM1 was particularly overexpressed in colorectal tumors

**Figure 3:**
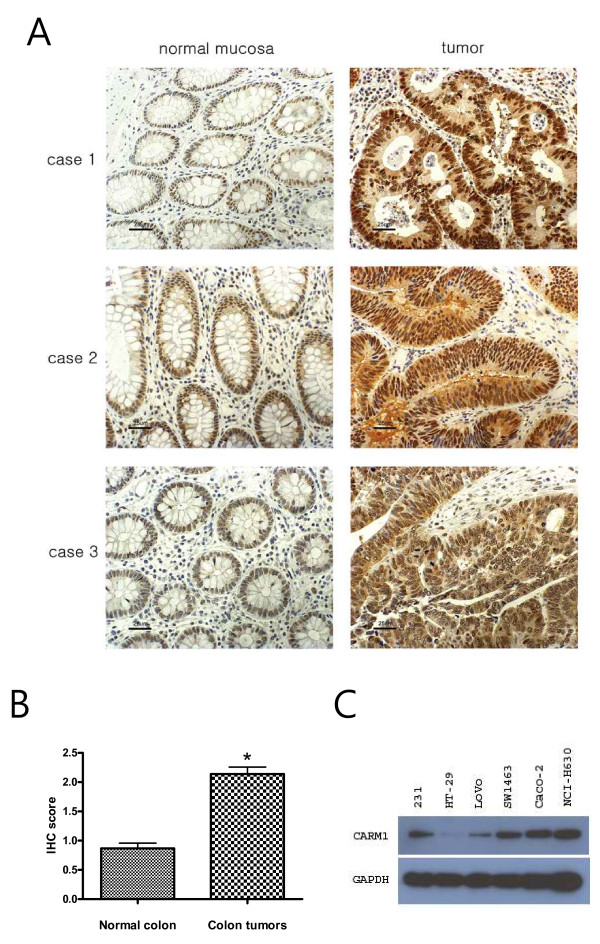
**Altered expression of CARM1 in colorectal cancers**. *A*, Immunolocalized CARM1 was observed in three cases of selected tumors with normal matching mucosa. Scale bar represents 25 μm. *B*, All tumors and normal mucosa were evaluated for their immunostained CARM1 intensity and statistical analysis was performed with the student's t-test *, p < 0.001; *C*, Western blot analysis for the expression of CARM1 in various colorectal cancer cells.

Since the tissue microarray showed that almost 70% of the colon cancers overexpressed CARM1, further studies were performed to confirm the expression of CARM1 in the colon cancers of surgically acquired specimens from the Indiana University Tissue Bank. These formalin-fixed and paraffin-embedded tissues included 65 colorectal tumors each with adjacent normal colonic mucosa. As shown in Figure 3A, CARM1 was highly expressed in some of the selected colon tumors while its expression was weak in the normal mucosal cells. Out of the 65 colorectal tumors, 49 expressed high levels of CARM1 (a score of 2-3), accounting for 75% of the total specimen. Overexpression of CARM1 in the colorectal tumors was statistically significant (student *t*-test p < 0.001) (Fig. 3B), confirming the CARM1 overexpression identified on the tumor tissue microarray. The Western blot analysis showed that CARM1 expression levels, in some selected colorectal cells, was high, and compatible with the MDA-MB-231 breast cancer cells, in most colon cancer cells except for HT-29. These findings suggested that CARM1 may play a role in non-hormone receptor dependent transcriptional regulation.

### CARM1's coactivating function for androgen activity was minimal in androgen-independent prostate cancer cells

**Figure 4:**
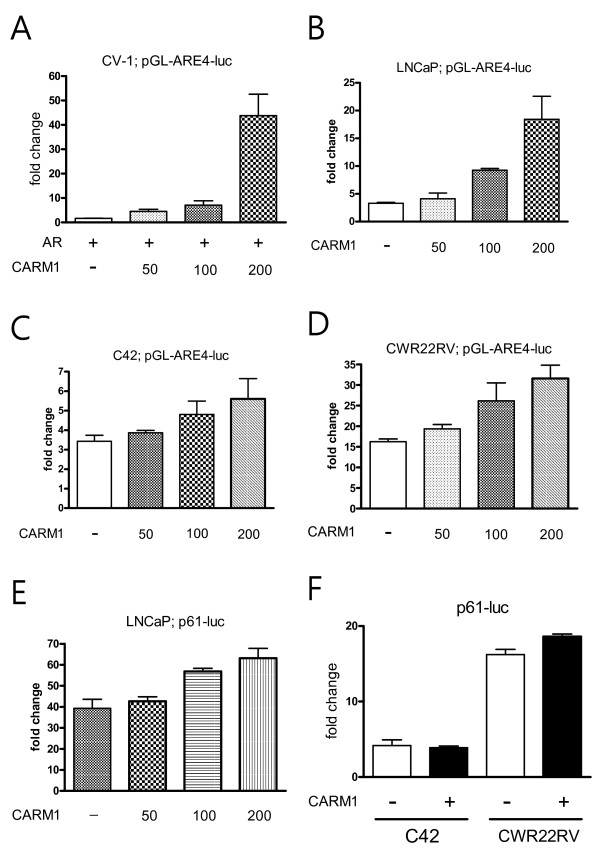
**Effect of CARM1 in androgen-stimulated androgen receptor mediated responses**. *A-D*, Androgen receptor (AR)-negative CV-1 cells, AR-positive LNCaP cells, and androgen-independent C42 and CWR22RV cells were transiently transfected with 100 ng of pGL-ARE4-luc, various amounts of pSG-CARM1, and 2 ng of renilla with or without 10 nM R1881. When needed, the pARO vector was cotransfected as indicated. *E-F*, the PSA promoter (p61-luc) was used, instead of pARE4-luc. Luciferase assays were performed 48 h post-transfection. Relative luciferase activity (RLU) was measured, and the hormone-treated group was normalized by the non-hormone-treated group. Values are represented as fold change. Each *bar *represents the mean ± S.D.

CARM1 is a histone methyltansferase, which primarily binds to histone and p160 coactivators to activate many nuclear receptors, including the estrogen receptor, glucocorticoid receptor, thyroid receptor, and androgen receptor [1,23]. Accordingly, CARM1 stimulates the promoter of the androgen-responsive prostate specific antigen in an androgen dependent manner [13]. To determine whether CARM1 plays a coactivating role in androgen-stimulated AR transactivation, various prostate cancer cells were studied in a reporter transcription assay. Using four copies of the synthetic androgen response element (ARE4), forced expression of CARM1 stimulated the androgen-activated ARE response with the cotransfection of AR in CV-1 monkey kidney cells (Fig. 4A). CARM1 also significantly activated ARE response in both AD LNCaP cells and AI C42 and CWR22rv cells (Fig. 4B). However, the coactivating effect of CARM1 was not significant in the C42 and CWR22RV cells, compared to the LNCaP cells (Fig. 4C-D). Moreover, using the entire PSA promoter spanning 5.9 kb, including the PSA promoter and enhancer region (p61-luc), CARM1's coactivating function with regard to the regulation of AR activity was minimal in the LNCaP cells (Fig. 4E). At the same time, androgen-independent PCa cells such as C42 and CWR22RV were not affected by CARM1 overexpression (Figure 4F). Since CARM1 has been reported to promote mouse mammary tumor virus (MMTV) promoter activity, which also contains ARE [24], these results were unexpected. CARM1 has also been reported to promote the PSA promoter [13]; although the reported promoter construct omitted -3872 to -542, which may contain a region involved in suppression. Therefore, these results suggest that CARM1 may affect other region(s) of the PSA promoter, while CARM1 generally showed coactivator function in AR transactivation.

### CARM1 exerts its effect by regulation of p53 and NF-κB responses

**Figure 5:**
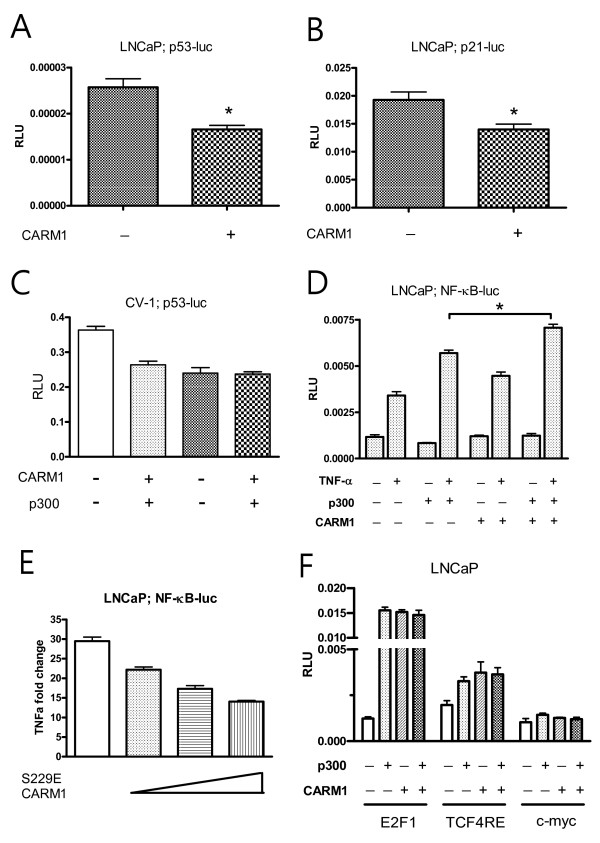
**Effect of CARM1 in p53 and NF-κB mediated signals**. LNCaP cells were transiently transfected with 100 ng of p53-luc (*A, C*), p21-luc (*B*), or NF-κB-luc (*D, E*) and 100 ng of pSG-CARM1 and 2 ng of renilla. In *C-E*, pRC/RSV-p300 was cotransfected. To activate the NF-κB response, TNF-α was added at a final concentration of 10 ng/ml after transfection; *E*, pSG-CARM1 (S229E) mutants were transfected up to 100 ng. *F*, E2F1-luc, TCF4RE-luc, and cmyc-luc were tested for the CARM1 response. Luciferase assays were performed 48 h post-transfection; each *bar *represents the mean ± S.D. *, p < 0.01.

**Figure 6:**
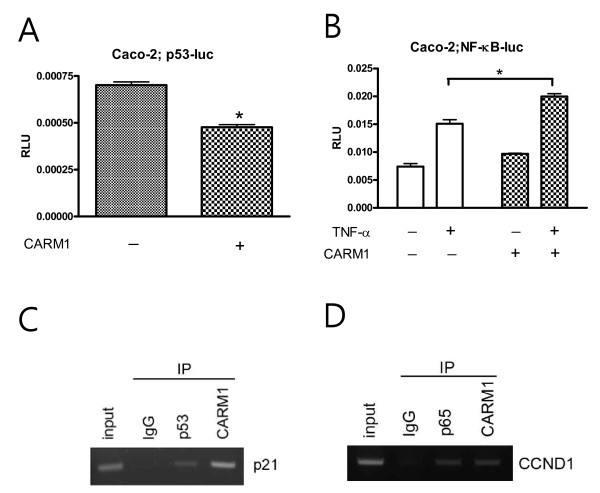
**In vivo association of CARM1 in p53 and NF-κB mediated signals**. Caco-2 colon cancer cells were also transfected with either p53-luc (*A*) or NF-κB-luc (*B*) with pSG-CARM1. CARM1 interacts in vivo with the promoters of p21 and cyclin D1 (CCND1). *C*, chromatin immunoprecipitation from caco-2 colon cancer cells using antibodies against p53, CARM1, and IgG. D, cells were grown in the presence of TNFα for 30 minutes and precipitated with antibodies against p65, CARM1, or normal IgG.

Other than the coactivator function of CARM1 in steroid receptors, CARM1 is a transcriptional activator of the p53 response [9] and the NF-κB-mediated response [25]. Therefore, we tested whether CARM1 promotes p53 target gene transcription in PCa cells. In contrast to previous reports suggesting that CARM1 activates the p53-mediated transcriptional response in H1299 lung cancer cells, CARM1 somehow inhibited the transcriptional activity of the p53 response elements (Fig. 5A) and subsequently the p21 promoter activity (Fig. 5B) (*p *values was 0.004 and 0.022, respectively). CARM1's transcriptional activation may require cooperation from the acetyl transferase p300 coactivator [9,25]. Cotransfection of p300 did not alter CARM1's inhibitory effects on the p53 response in the CV-1 monkey kidney cells (Fig. 5C). CARM1, however, promoted the NF-κB-mediated transcriptional response as reported previously (Fig. 5D) [25]. Cotransfection of p300 was additive with regard to the effects of CARM1. Blocking endogenous CARM1 using S229E CARM1 mutants showed significant inhibition of TNFα-stimulated NF-κB activity (Fig. 5E). However, CARM1 did not affect the activities of other promoters, including E2F1, TCF4 response elements, and c-myc (Fig. 5F). To determine whether CARM1 regulates p53 and NF-κB responses in colon cancer cells, we used CARM1-positive Caco-2 and CARM1-negative HT-29 cells for the luciferase assay. While HT-29 did not provide consistent data, due to the poor transfection efficiency with the liposome (data not shown), CARM1 inhibited the p53 response and promoted the NF-κB response in the Caco-2 cells (p < 0.01) (Fig. 6A-B). Correspondingly, chromatin immunoprecipitation assay demonstrated that CARM1 is associated with promoters of p21 (Fig. 6C) and cyclin D1, a target of NF-κB-mediated transactivation (Fig. 6D). These results suggest that CARM1 exerts a biological effect, as yet unknown, by regulation of p53 and NF-κB target gene expression, especially in colon cancer cells and androgen-independent prostate cancer cells.

## Discussion

Aberrant expression of CARM1 has been suggested to be important in tumors of the breast and colon [7,11-13]. Our study, however, demonstrated that CARM1 was mainly overexpressed in colorectal tumors; with a frequency as high as 68% (Table 1), compared to other tumors from the brain, skin, ovary, lymphocytes, lung, breast, and prostate. CARM1 overexpression was noted in some hormone-dominated tumors such as breast cancer (27%) and ovarian cancer (17%), and in only 6% of prostate cancers. Considering the interest in the role of CARM1 in hormone receptor modulation these frequencies were lower than expected. These findings suggest that CARM1 may act by other transcription events for which there is limited information.

Follow-up studies with surgical specimens further demonstrated that CARM1 was commonly expressed in over 75% of colorectal tumors (49 out of 65 tumors expressed high levels of CARM1). Previously, CARM1 was shown to be overexpressed in both androgen-sensitive tumors and androgen-independent tumors with increased expression in castration-resistant prostate cancers [12]. In a more recent study, CARM1 was not found to be generally overexpressed in androgen sensitive tumors, but has been suggested to play a role in androgen independent progression of prostate cancer [13]. In this study, however, the CARM1 expression was low in most prostate cancers, including both androgen-sensitive and androgen-resistant tumors. The explanation for this difference between studies is not clear. However, one can assume that acquired hormone-resistant tumor samples are quite different depending on the treatment protocol from various institutions. This is especially true considering that acquisition of real hormone dependent tumors is difficult since most patients at this stage fail to respond to hormone ablation therapy. In cultured PCa cells, there was low expression of CARM1 in most androgen-dependent PCa cells while high expression of CARM1 was observed only in androgen-independent PC3 and DU145 cells (Fig. 2A-B). While these cells are described as androgen-independent PCa cells, they are generally considered as non-prostate-like cancer cells mainly due the deficiency of androgen receptors and thereby lack of PSA expression. In cultured cancer cells, mRNA and protein level of CARM1 were not always correlated, especially in 231 breast cancer cells and CWR22RV PCa cells. This discrepancy is not clearly understandable. Ohkura et al have demonstrated that there are at least one other isoform of CARM1 existed in endogenous cell level by alternative splicing [26]. This isoform may have contributed to increased CARM1 protein expression compared to the level of RNA. It is also noteworthy that, in addition to its transcriptional coactivator role, CARM1 also regulates target gene expression by modulating protein stability, including p/CIP [27], AP-1 [28], and NF-κB [29]. Taken together, our conclusions imply that CARM1 is not overexpressed in hormone refractory prostate cancers well correlate with this phenomenon.

CARM1 functions as a coactivator for many nuclear receptors, such as the estrogen receptor (ER), AR, the glucocorticoid receptor, and the thyroid receptor [1,3,10]. For example, CARM1 has been implicated in the positive regulation of ERα-mediated gene activation in response to estrogen signaling [30]. Most nuclear receptors, including ARs, are bound directly by p160 coactivators whose N-terminal region interacts with CARM1 to transmit the activating signal to transcription machinery [10,30]. In fact, CARM1 exerts an effect on methyltransferase activity to mediate this process [10]. CARM1 functions as a coactivator of the AR to activate target promoters containing androgen response elements, such as MMTV, PSA, and probasin [10,13]. Although in this study we observed the same phenomenon while we used a simple ARE construct in LNCaP cells, a strong coactivating function of CARM1 was not detected in most androgen-independent cells, including C42 and CWR22RV. Moreover, using the entire PSA promoter spanning 5.9 kb in the PSA promoter and enhancer region, CARM1 did not significantly increase promoter activity, especially in C42 and CWR22RV cells. Majumder et al demonstrated that CARM1 exerted strong coactivating effects on androgen-stimulated AR activity; however, they used a PSA promoter region containing only -5,322 to -3,873, which may explain the different findings in the studies [13]. Nevertheless, our results suggest that regulation of PSA expression in PCa cells by CARM1 was mainly mediated by other transcription factors transactivating the PSA promoter region.

CARM1 also coactivates other transcription factors such as the myocyte enhancer factor 2C (MEF2C), p53, NF-κB, and E2Fs [7-10], suggesting that CARM1's effect may not primarily be associated with the AR function in PCa cells [13]. The results of this study demonstrated that forced expression of CARM1 alone slightly inhibited the activity of the p53-response, in contrast to the previous report by An et al [9]. CARM1 has also been reported to exert coactivator effects on the GADD45 promoter, another p53 target gene, in p53-deficient cells [9]. The reason for this difference is not clear. However, CARM1 may provide coactivator activity in a tissue-specific manner. In addition, we showed that CARM1 can activate the NF-κB response by stimulation of TNF-α. Both NF-κB and p53 pathways are key mediators of genes involved in the control of the cellular proliferation and apoptosis [31,32]. Antiapoptotic genes that are directly activated by NF-κB include c-IAP1, c-IAP2, and IXAP, TNF receptor-associated factors, the Bcl-2 homologue A1/Bfl-1, and IEX-IL [33]. NF-κB directly induces expression of A1/Bf1-1 by binding to specific sites in its promoter [34]. NF-κB also acts in the control of the cell cycle. NF-κB activates the expression of cyclin D1, a positive regulator of G1-to-S-phase progression, by direct binding to multiple sites in its promoter [35]. Thus, the apoptotic regulation by either NF-κB or p53 involves the regulation of multiple genes involved in different aspects of growth control. Therefore, the results of this study provide evidence that CARM1 may exert enzyme effects to non-hormone receptor, transcriptional regulatory molecules, such as p53 and NF-κB to modulate the target gene expression and subsequently the proliferation of colon cancer cells. At the same time, malignant progression of prostate cancer might be achieved by androgen-independent activity of CARM1.

## Conclusions

In the field of steroid receptor-mediated transcriptional regulation, CARM1 functions as a coactivator that confers steroid receptor-mediated transactivation. The results of this study demonstrated that expression of CARM1 was especially high in colorectal cancers, over 75%. In contrast to previous reports, CARM1 was not overexpressed in the androgen sensitive and androgen-resistant prostate cancers. In addition, CARM1 modulated the transcription activity of p53 and NF-κB. These results suggest that CARM1 may exert its methylase activity on non-hormone receptor-related transcriptional machinery to modulate the biology of cancer cells, including androgen-independent prostate cancers and colorectal cancers.

## Abbreviations

CARM1: Coactivator-associated arginine methyltransferase 1; PSA: prostate-specific antigen; NF-κB: Nuclear factor-kappa beta; AR: androgen receptor; ARE: androgen response element; CDT: charcoal-dextran treated; MMTV: mouse mammary tumor virus; AD: androgen dependent; AI: androgen independent; ER: estrogen receptor; MEF2c: myocyte enhancer factor 2c.

## Competing interests

The authors declare that they have no competing interests.

## Authors' contributions

Y-RK: carried out the majority of experiments; BKL: analyzed immunohistochemical data and wrote the manuscript; R-YP: supported with expertise in molecular biology techniques and in data preparation; NTXN: carried out immunohistochemical experiments; JAB carried out chromatin immunoprecipitation assay; DDK: participated in preparation of prostate cancer and data interpretation; CJ coordinated and designed the study, interpreted data, and drafted the manuscript. All authors read and approved the final manuscript.

## Pre-publication history

The pre-publication history for this paper can be accessed here:

http://www.biomedcentral.com/1471-2407/10/197/prepub
